# Evaluation of the Antibacterial Activity of New Dermaseptin Derivatives against *Acinetobacter baumannii*

**DOI:** 10.3390/ph17020171

**Published:** 2024-01-29

**Authors:** Houda Haddad, Radhia Mejri, Amira Zaïri

**Affiliations:** 1 BIOLIVAL Laboratory, LR14ES06, The Higher Institute of Biotechnology of Monastir ISBM, University of Monastir, Monastir 5000, Tunisia; haddadhouda1993@gmail.com; 2 Biochemistry Department, LR18ES47, Faculty of Medicine, University of Sousse, Sousse 4002, Tunisia; mejriradhia116@gmail.com

**Keywords:** dermaseptin B2, dermaseptin S4, analogs, *Acinetobacter baumannii*, healthcare-associated infections, antibacterial activity

## Abstract

Nosocomial infections represent one of the biggest health problems nowadays. *Acinetobacter baumannii* is known as an opportunistic pathogen in humans, affecting people with compromised immune systems, and is becoming increasingly important as a hospital-derived infection. It is known that in recent years, more and more bacteria have become multidrug-resistant (MDR) and, for this reason, the development of new drugs is a priority. However, these products must not affect the human body, and therefore, cytotoxicity studies are mandatory. In this context, antimicrobial peptides with potential antibacterial proprieties could be an alternative. In this research, we describe the synthesis and the bioactivity of dermaseptins and their derivatives against *Acinetobacter baumannii*. The cytotoxicity of these compounds was investigated on the HEp-2 cell line by MTT cell viability assay. Thereafter, we studied the morphological alterations caused by the action of one of the active peptides on the bacterial membrane using atomic force microscopy (AFM). The cytotoxicity of dermaseptins was concentration-dependent at microgram concentrations. It was observed that all tested analogs exhibited antibacterial activity with Minimum Inhibitory Concentrations (MICs) ranging from 3.125 to 12.5 μg/mL and Minimum Bactericidal Concentrations (MBCs) ranging from 6.25 to 25 μg/mL. Microscopic images obtained by AFM revealed morphological changes on the surface of the treated bacteria caused by K_4_S4(1-16), as well as significant surface alterations. Overall, these findings demonstrate that dermaseptins might constitute new lead structures for the development of potent antibacterial agents against *Acinetobacter baumannii* infections.

## 1. Introduction

Healthcare-associated infections (HAIs), also known as nosocomial infections, are infections acquired within a healthcare establishment which were not initially present or incubating at the time of patient admission. They generally manifest 48 h or more after hospitalization [1]. HAIs can be transmitted via patient contact with healthcare personnel, contact between patients, medical devices, procedural interventions by means of hand contact, or oral, parenteral, aerial or vector-borne transmission [2]. These infections are caused by various microorganisms, such as multidrug-resistant bacteria (MDR) and other agents responsible for their occurrence. World Health Organization (WHO) data estimate that HAIs lead to approximately 40,000 deaths per year. These nosocomial infections have rates of up to 25% in developing countries; in contrast, developed countries show values of around 5 and 15% [3,4].

*A. baumannii*, a Gram-negative coccobacillus, is the predominant human pathogen in hospital settings and presents considerable viability on human hands, contributing to significant cross-contamination rates in nosocomial infections [5]. Nowadays, *A. baumannii* holds the highest rank on the WHO priority pathogen list (“critical”), highlighting its importance as a nosocomial causative pathogen, particularly in cases where it presents resistance to carbapenem, the “last-resort” antibiotic [6]. A previous study reported that *A. baumannii* is an agent that is responsible for ocular infections. Ophthalmic complications in burn victims have been observed due to corneal injuries or an associated trauma. *Acinetobacter* ophthalmic infections are more common in patients requiring respiratory assistance [7]. Therefore, this multidrug-resistant bacillus is the top priority in antibiotic research and development efforts due to the ineffectiveness of antibiotics. The evolution of the antimicrobial resistance of *Acinetobacter* strains to antibiotics began in 1932 [8]. These antibiotics include beta-lactams [9], carbapenems [10], colistin [11], tigecycline [12] and quinolones [13]. 

For years, the medical field has witnessed the emergence of new resistance mechanisms in *A. baumannii* against antimicrobial treatments, leading to a reduction in the effectiveness of therapeutic interventions. However, to our knowledge, none of these investigated candidates has progressed beyond preclinical evaluation [14]. For these reasons, the development of new drugs for HAIs caused by *A. baumannii* is required urgently, preferably employing compounds effective in low concentrations which are non-toxic and selective against this bacterium. Cationic antimicrobial peptides (AMPs), especially dermaseptins, could be candidates for such assays. AMPs are small peptides, composed of 5 to 100 amino acid residues, which present a wide range of molecular weights (<10 kDa) [15,16]. Various organisms, such as plants, insects, amphibians, and mammals, produce these peptides through their innate immune system; they are derived from various sources such as macrophages, neutrophils, epithelial cells, hemocytes, fat bodies, reproductive tracts, etc. [17].

Among these, AMPs are dermaseptins (DRSs), a family identified in the skin of the South American *Phyllomedusa* frog. DRSs are secreted by amphibians’ skin as part of their defense mechanism against microbes and are generally composed of 28–34 amino acids, exhibiting significant variability among different peptides. Nevertheless, in apolar solvents, they tend to adopt amphipathic α-helices [18]. DRSs exhibit substantial variation in both their peptide sequences and lengths. However, they share structural similarities, such as the presence of conserved Trp residue at position 3, a conserved sequence of AA(G)KAALG(N)A in the middle region, and a net positive charge [19]. Overall, DRS-S peptides isolated from the secretions of *Phyllomedusa sauvagii* have not been widely used in various human antimicrobial applications. These applications are particularly interesting due to their ability to effectively eliminate microbes without encountering resistance and their rapid mechanism for killing pathogens [20]. So far, a total of thirteen DRSs (DS-1 to 13) have been discovered and characterized [21]. 

Based on existing data regarding the action of dermaseptins against bacteria, we can identify two distinct types of mechanisms. The initially proposed mechanism involves the “barrel-stave” model; it is mediated by the attachment of DRSs to membrane phospholipids. This interaction disrupts the osmotic balance of the cell, leading to membrane permeabilization. Subsequently, transmembrane pores or channels form, eventually causing membrane rupture. The second mechanism, called the “carpet-like” mechanism, involves the binding of positively charged lytic peptides to the negatively charged surface. This method of destruction allows complete surface coverage, leading to the impregnation and disintegration of the membrane [22]. 

Certain DRSs exhibit an impressive ability to efficiently, rapidly, and irreversibly inhibit microbial cells without causing toxicity in mammalian cells [20]. Besides their large spectrum of activity, DRSs are not hemolytic [21], except for DRS-S4, which demonstrates a potent hemolytic effect [23]. In the same context, DRS-B2, from *Phyllomedusa bicolor* [24], is alternatively referred to as adenoregulin due to its capacity to modulate the binding of adenosine A1 receptor agonists [25]. Among the peptides of the DRS family, DRS-B2 stands out, with the highest level of activity, making it the subject of extensive research. This amphipathic (+3) cationic polypeptide is composed of 33 amino acids, with a molecular weight of approximately 3180 Da, a tryptophan residue at position 3, and six lysine residues [26]. This α-helical peptide has the ability to disrupt the membranes of a wide range of microorganisms, such as bacteria, yeast, fungi, and protozoa. However, its specific mechanism of action remains unclear [27]. 

To our knowledge, no studies have been carried out to evaluate the antibacterial effect of the native B2 and derivatives from both DRS families against *A. baumannii*. Thus, in the current research, we report, for the first time, the in vitro antibacterial activity of these peptides against *A. baumannii*. DRS derivatives were synthesized, purified, and evaluated for their antibacterial activity. Their cytotoxicity toward HEp-2 cells was evaluated, and then atomic force microscopy (AFM) was performed to describe morphological changes in the bacteria.

## 2. Results and Discussion

### 2.1. Design of Dermaseptin S4 and B2 Derivatives

In our study, the peptides were designed based on three main steps of structural modifications: substitutions and/or deletions to S4 and B2 sequences (Table 1). K_4_K_20_S4 is a derivative that was formed by replacing methionine (M) in position 4 and asparagin in position 20 with a lysine (K) and designating it as M4K and N20K. Peptides K_4_S_4_(1-16) have the same M4K substitution, with the deletion of 12 C-terminal residues. The K_3_K_4_B2 derivative was obtained by a double substitution, i.e., a tryptophan residue (W) with a lysine (K) at position 3(W3K) and a serine residue (S) with a lysine at position 4 (S4K) of B2. The one-letter code was used to identify the amino-acid sequence of these peptides, taking as reference the sequence and length of native peptides DRS-S4 and DRS-B2 (Table 1).

The first modification step was intended to increase the hydrophilic characteristics and decrease hydrophobicity. Since S4 and B2 exhibited some levels of functional activity with cationic residues, our initial focus in modifying this peptide was to enhance its hydrophilic properties by adding basic amino acids, such as lysine. The choice of lysine was also made to prevent an increase in cytotoxicity. In fact, it was previously shown that increasing the net positive charge and decreasing the hydrophobicity of DRS-S4 led to decreased hemolytic activity while maintaining high biological activity [23,28,29]. The S4 sequence may be minimized by truncating 12 amino acids in the secondary structure. It has been reported that the use of AMPs as therapeutic agents faces challenges, attributed to their long peptide sequences, limited efficacy, instability, systemic toxicities, and the potential to compromise the innate defense immunity of the host. These factors have hampered the progress and clinical implementation of AMPs [30]. Several approaches have been applied in the design of analogous peptides to surmount these obstacles. These methods include motif hybridization, aimed at enhancing antimicrobial efficacy and functionality [31], truncation/substitution intended to reduce toxicity [32], and de novo design, aimed at shortening peptide length and eliminating host defense immunogenicity [33]. Previous data suggested that the N-terminal domain of DRS demonstrates selectivity during interactions with the bacterial cell membrane, while the C-terminal helix mainly exhibits nonspecific membrane lytic activity [34,35]. Previous studies on the N-terminal peptide fragments of DRSs revealed that truncated peptides ranging from 16- to 19- mer maintain comparable antimicrobial activity. However, shorter sequences, i.e., containing less than 13 amino acid residues, experience a significant reduction in antimicrobial activity [36,37]. In summary, the criteria selection of peptides was examined as a means of defining the structural requirements for biological activity. Regarding B2, to the best of our knowledge, the present research was the first time that the derivative K_3_K_4_B2 had been used. Indeed, modifications made to the native B2 molecule primarily involved truncation, such as the design of a C-terminal truncated analog known as [1-23]-DRS-B2. Despite maintaining the net cationic charge of the native peptide B2, this truncated analog was found to be inactive against bacteria [27]. Combining DRS-B2 with alginate nanoparticles (Alg NPs) results in a formulation (Alg NPs + DRS-B2) that creates novel B2 derivatives displaying significant antibacterial efficiency against both susceptible and resistant strains of *Escherichia coli* to colistin. The antibacterial activity achieved with this innovative formulation exceeds that of DRS-B2 when used alone [26]. Moreover, our synthesized peptides offer the advantage of being configured with D-amino acids. In contrast, peptides in their natural form consist of L-amino acids, which pose a challenge due to their susceptibility to degradation by proteases, thereby limiting their clinical applications [38]. As previous research pointed out, a feasible approach to address these constraints involves replacing the L-amino acids at the most susceptible site with D-amino acids [39]. Obviously, substituting with D-amino acids does not alter the net positive charge of the native peptide; however, it does impact the structure and function associated with recognizing chiral targets [40,41]. 

### 2.2. Structural and Physicochemical Properties of Peptides

**Figure 1:**
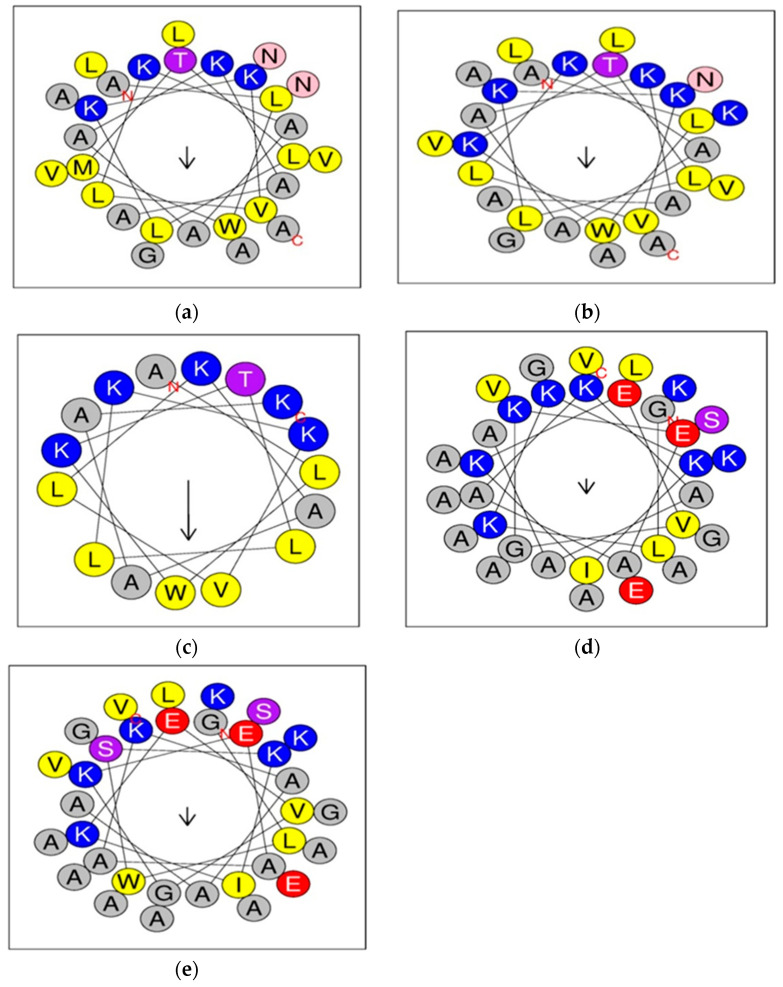
Helical structure of dermaseptines and their derivatives. These peptides are represented as a two-dimensional axial projection of an ideal α-helix. (**a**) Helical structure of the native S4; (**b**) Helix structure of K_4_K_20_S4; (**c**) Helical structure of K_4_S4(1-16); (**d**) Helical structure of K_3_K_4_ B2; (**e**) Helical structure of the native B2. The figures use the one-letter code for amino acids. The aminoacids are presented with different colors according to their proprieties: (grey: nonpolar residue; blue: positively charged residue; yellow: hydrophobic residue; red: for acidic; pink: for Asn (N); purple: The Thr (T) and Ser (S); the arrow in helical wheels corresponds to the hydrophobic moment μH). Figure built using Heliquest software ComputParams form version 3 [28].

According to Heliquest, we noted that the K_4_K_20_S4 peptide had the highest value of Hydrophobicity (H) among the S4 peptide derivatives (0.451), whereas K_4_S4(1-16) had the lowest value (0.426) (Table 1). In general, all peptide derivatives of DRS-S4 had a lower H than the native peptide (S4). This was also confirmed for DRS-B2 and its derivative, where the H value was higher (0.199) than the K_3_K_4_B2 value (0.072). This hydrophobicity is a crucial physicochemical characteristic of peptides. Typically, it is determined by analyzing the peptide sequence [42]. It plays an important role in their conformational modifications, stability, and molecular interactions [43]. The μH ranged from 0.159 to 0.526, where K_4_S4(1-16) showed the highest value (μH = 0.526) and K_3_K_4_B2 showed the lowest (μH = 0.159) (Table 1). The hydrophobic moment μH is established through the calculation of the vector sum of the hydrophobicity values for each individual amino acid [44,45]. This factor significantly influences the interfacial binding of peptides with the membrane [46]. Based on the TANGO algorithm [47], we noticed that DRS-S4 presented a greater tendency to aggregate than its derivatives due to the presence of two N-terminal and C-terminal hydrophobic domains (Table 1, Figure 1). These results agreed with those of Feder et al., who demonstrated that aggregation in DRS-S4 and its derivatives occurs through hydrophobic interactions. Indeed, the M_4_KN_20_K substitution in the N-terminal and C-terminal domains induced a decrease in the aggregation tendency of K_4_S4, which was in accordance with other studies [23,48]. However, the deletion of the C-terminal domain of S4 and the insertion of positive charges in these regions resulted in a loss of aggregation. K_4_(1-16)S4 had a value of aggregation equal to zero (Table 1). Hence, the deletion of the hydrophobic domains and/or the insertion of positive charges in these regions will probably decrease the aggregation, either due to the absence of such hydrophobic domains or by electrostatic repulsion between cationic residues [23,49]. These results revealed that the aggregation of peptides is influenced not only by their hydrophobicity, but also by their charge distribution. Thus, the introduction of cationic amino acids to one of the hydrophobic domains, and/or the elimination of hydrophobic domains, adversely affects aggregation. Regarding DRS-B2, we noticed that neither substitution nor deletion induced a loss or a decrease of aggregation since the native molecule had a very low degree of aggregation (9.681) comparing to S4 (Table 1). Previously, it has been demonstrated that the tendency to aggregation in aqueous solution is another important parameter for antibacterial activity and cell selectivity [50]. This property can be explained by the ability of peptides to form aggregates (oligomers) in aqueous solution, establishing hydrophobic interactions with other monomers. Due to the increase in size and the loss of flexibility, such aggregates are unable to pass through polysaccharide capsules, outer membranes, or bacterial cell walls, making them unable to interact with the plasma membrane, which is the action target. Consequently, peptides with a high tendency for aggregation are weakly active against bacteria; on the other hand, for a monomeric peptide, it is easier to reach the plasma membrane and undertake effective antibacterial activity [51]. Therefore, in designing antimicrobial peptides, it is usually desirable to reduce aggregation in order to favor antibacterial activity [29,51]. In our study, we noticed that the derivatives of all of our peptides had reduced aggregation, which would provide them with potential biological activity, such as antibacterial activity.

To estimate the helicity of the studied peptides, we used the AGADIR algorithm [52], which indicated that native molecule S4 had the greatest helicity (as α-helix%), i.e., approximately eightfold greater than K_4_S4(1-16) and similar to that of K_4_K_20_S4. However, DS-B2 and its derivative had the same helicity (10.2% and 9.85% respectively) (Table 1). It has been reported that helicity is assessed to investigate the relationship between the secondary structure and selectivity against microbial cells of α-helical antimicrobial peptides [53]. Therefore, the high potency of the dermaseptins may be attributed not only to a higher net positive charge, but also to the stabilization of the helical conformation [54]. 

### 2.3. In Vitro Toxicity of Dermaseptin and Derivatives against HEp-2 Cells

All our peptides were evaluated for their cytotoxicity on HEp-2 cells using the MTT viability assay; the results are shown as survival rates after 72 h of treatment with the compounds (Table 2). 

Considering our results, the tested cationic derivatives exhibited variable toxicity toward HEp-2 cells. Cells were exposed to increasing concentrations of peptides, ranging from 1.5 to 100 μg/mL. The cytotoxicity of DRSs was concentration dependent, and peptide 50% cytotoxic concentration (CC_50_) values were determined (Table 2). The highest cytotoxicity values for all S4 and B2 derivatives were recorded at concentrations higher than 61.25 μg/mL (CC_50_). Interestingly, our results showed that shortening the peptide in the C-terminal extremity of K_4_S4(1-16) resulted in a CC_50_ equal to 68.9 μg/mL, and increasing its positive charge with different substitutions (K_4_K_20_S4 or K_3_K_4_B2) yielded peptides with low toxicity, with CC_50_ of about 75.71 μg/mL and 61.25 μg/mL, respectively. Belaid et al. [55] demonstrated that the maximal non-cytotoxic concentrations were 32 μg/mL for DRS-S1 and DRS-S2, 16 μg/mL for DRS-S3 and DRS-S4, and 64 μg/mL for dermaseptin S5 to HEp-2 cells. Additionally, the study of Gourkhede et al. [56] indicated that other AMPs, like Cecropin A (1–7)-Melittin and lactoferricin (17–30), exhibited negligible cytotoxicity at lower concentrations (1X and 2X MIC); however, at 4X MIC, slightly higher cytotoxicity was observed, given that the MIC values were equal to 64 μg/mL and 128 μg/mL, respectively. Sruthy et al. [57] confirmed that at the highest tested concentration of histone H2A-derived antimicrobial peptide (200 μM), growth inhibition of 89% was observed in HEp2 cell lines. Another study conducted by Hazime et al. [26] tested the cytotoxic effects of DRS-B2 and of a new formulation (Alg NPs + DRS-B2) on the human erythrocytes and eukaryotic cell line types HT29 (human) and IPEC-1 (animal); in that study, their safety was verified. Zairi et al. [58] found that dermaseptin K_4_S4 exhibited an enhanced toxicity profile against human endometrial epithelial cells, displaying lower sensitivity to the toxic effects of dermaseptins compared to other cell types. While dermaseptin S4 and its derivatives have demonstrated significant cytotoxicity against the SW620 cell line, it remains challenging to discern their cellular selectivity or mode of action [59]. Moreover, Lorin et al. [60] demonstrated that dermaseptin K_4_S4(1-16)a exhibited a comparable effect on both HeLa P4-CCR5 cells and primary PBMCs, no toxicity in mice, and reduced cell toxicity at high concentrations. All this work has shown that synthesized and modified peptides are less toxic to HEp-2 cells compared to native molecules S4 and B2.

### 2.4. Antibacterial Activity of Dermaseptin Derivatives against Acinetobacter baumannii

The susceptibility of bacteria to DRS-S4 and DRS-B2 derivatives was assessed by measuring the peptide MIC against the clinical isolate, i.e., *A. baumannii*, a MDR gram-negative bacteria. The resulting data are summarized in (Table 2). The data show that all peptides tested inhibited *A. baumannii* growth and that this was dependent on the nature of the peptide, with highly charged molecules being the most active. The MICs ranged from 3.125–12.5 μg/mL. These findings demonstrate the potential of these peptides as antibacterial agents. Thus, in Table 2 we can notice that the most effective peptide is K_4_K_20_S4; this peptide displayed potent antibacterial activity, with MICs of about 3.125 μg/mL. Likewise, the mono-substituted truncate peptide K_4_S4(1-16) presented a nearly homogenous potency, with a MIC value equal to 6.25 μg/mL. The truncation of the C-terminal extremity resulted in a less toxic peptide and did not affect its potency. As Kustanovich suggested, interaction with the cell membrane of the N-terminal domain mainly depends on the net charge state, while the C-terminal domain also contributes to the binding affinity [29]. The native DRS-S4 has a MIC of 12.5 μg/mL and was the less potent against *A. baumannii*, but S4 presents the same MICs value as the native B2. The analog K_3_K_4_B2 is likewise more active compared to the native B2; the peptide exhibits a MIC equal to 6.25 μg/mL (same value as K_4_S4(1-16)). The increase of the positive charge for the derivatives of both of DRS-S and DRS-B is essential to increase their antibacterial activities. Therefore, K_4_K_20_S4 and K_3_K_4_B2 are the most active analogs against *A. baumannii*, since they have the lowest MIC values. In the same context, our result showed that native molecules such as B2 and S4 remain less active compared to their cationic analogs.

After determining the MIC, the MBC was assessed against *A. baumannii* MDR. Meropenem was used as an antibacterial control drug. The loaded solvent saline served as a negative control. However, meropenem, which was used as a reference, presented an MIC of 32 µg/mL and an MBC value of 64 µg/mL. Meropenem, belonging to the carbapenem class of beta-lactam antibiotics, exhibits a broad spectrum of activity and minimal toxicity. This antimicrobial agent provides effective coverage against various microorganisms, making it a valuable and frequently prescribed treatment for the management of severe and nosocomial infections [61]. Meropenem targets Gram-positive and Gram-negative bacteria, as well as anaerobic bacteria. Similar to other carbapenems, meropenem disrupts the synthesis of bacterial cell walls, inhibiting growth and leading to cell death [62]. Inadequate concentrations of meropenem may result in treatment failure and increase the risk of microbial resistance emergence [63]. 

MBC showed results equal to or greater than the MIC values, with values ranging from 6.25 μg/mL to 25 μg/mL for both dermaseptins derivatives. The highest MBC (25 μg/mL) was observed with native molecules S4 and B2. The lowest MBC (6.25) was for K_4_K_20_S4. For K_4_S4(1-16) and K_3_K_4_B2, MBC was 12.5 μg/mL. However, it has been reported in a previous study of Jiang et al. [64] that some dermaseptins, i.e., S4 and Piscidin 1 and their derivatives (piscidin 1 (I9K), as well as dermaseptin S4 (L7K, A14K)), were active against the Gram-negative pathogen *A. baumannii* (11 strains). In the case of D-dermaseptin S4, the geometric mean of MIC values for *A. baumannii* decreased from 1.8 µM for D-dermaseptin S4 to 1.1 µM for D-dermaseptin S4 L7K, A14K, indicating a small improvement in antimicrobial activity [64]. D-dermaseptin S4 L7K, A14K, is the most selective peptide of the dermaseptin S4 analogs. For *A. baumannii*, the therapeutic index improved by 730-fold, from 0.3 for native D-dermaseptin S4 to 219 for this analog [64]. To the best of our knowledge, this is the first time that the antibacterial activity of DRS-B2 and its derivative against *A. baumannii* have been evaluated. A previous study demonstrated that DRS-B1 and S1 exhibit in vitro activity against both Gram-positive and Gram-negative bacteria, demonstrating varied specificities [65]. Furthermore, the literature shows that derivatives of DRS-S4, DRS-CA1, DRS-DU1, and DRS-PH present in vitro activity against *Staphylococcus aureus* (including the methicillin resistant strain), *Pseudomonas aeruginosa,* and *E. coli*, even when they are formed in a biofilm [36,66,67,68]. In research by Zairi et al. [66], DRS-S4 derivatives were shown to be less cytotoxic than conventional antibiotics [66].

Understanding the peptide–membrane interaction and resistance mechanisms of *A. baumannii* is crucial for the development of new antimicrobial agents or alternative tools to combat this public health challenge. The mechanisms of drug resistance can be broadly categorized into several groups, such as drug inactivation or alteration, modification of drug binding sites or targets, alterations in cell permeability leading to decreased intracellular drug accumulation, and the formation of biofilms [69]. AMPs are known for their primary interaction with the bacterial cytoplasmic membrane, affecting both membrane integrity and electrical potential [70]. Gram-negative bacteria consist of two layers of membranes; at the moment of interaction, AMPs must first penetrate the outer membrane before exerting their effects on the cytoplasmic membrane or further acting on the bacteria [71]. While interacting with the membrane, AMPs might additionally influence the trans-membrane voltage, referred to as the membrane potential, which typically regulates ATP synthesis, membrane transport, and cell division [71]. On the other hand, Jiang et al. [64] suggested that AMPs interact with negatively charged prokaryotic cell membranes by employing a detergent-like mechanism (also known as the carpet mechanism) [72], where antimicrobial activity does not necessitate trans-membrane insertion [64]. Cationic peptides can align parallel to the bacterial membrane surface, where the positively charged residues on the polar face interact with the negatively charged phospholipid headgroups of the bilayer, and the ε-amino group of the Lys side chain of the specificity determinant(s) can be long enough to prevent hydrophobicity of the bilayer when lying parallel to the membrane surface, even if they are on the nonpolar face of the AMPs. These peptides conserve their ability to disrupt the lipid bilayer, leading to cytoplasmic leakage and cell death [64].

Our study also aimed to improve the antibacterial activity of our peptides and has focused on morphological alterations due to the interaction of our modified peptide K_4_S4(1-16) with the outer membrane of *A. baumannii*.

### 2.5. The Morphological Effect of K_4_S4(1-16) on the Treated Bacteria

**Figure 2:**
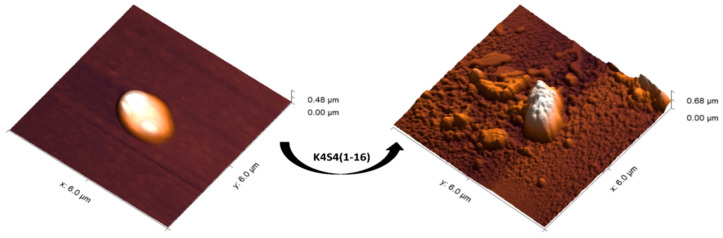
AFM images of *A. baumannii* before and after treatment with the K_4_S4(1-16)peptide.

In order to understand how our cationic peptide eliminates bacteria, we must consider its effect on the outer membrane found in Gram-negative bacteria. Here, we use atomic force microscopy (AFM) to directly investigate K_4_S4(1-16) interactions with the outer membrane of *A. baumannii* and characterize the biophysical consequences of K_4_S4(1-16) treatment. However, Gram-negative bacteria are complex organisms, with two membranes and molecular machinery dedicated to maintaining membrane stability. The images show the morphology of a clinical strain of *A. baumannii* before and after treatment with the derivative. AFM imaging revealed that the membrane had become pitted, more flexible, and more adhesive after K_4_S4(1-16) treatment (Figure 2). It also showed changes in the appearance in the cell envelope of the treated bacteria; the changes became more pronounced, including more variable cell shape with shrinkage and membrane disruption. The cell differed considerably from the cocci-bacilli-shaped cell observed without peptide treatment. Therefore, this cationic peptide appears to have a highly disruptive effect. This interaction caused changes in elasticity and adhesion, as well as increased roughness on the cell surface after treatment, i.e., from 25.77 nm to 43.41 nm, compared to the smooth surface of the non-treated bacteria. 

The changes observed during AFM may have been caused by a disruption of membrane integrity, which could lead to cell osmolarity without the occurrence of lysis, as shown in the presented images [73]. Few studies have focused on the membrane changes observed in treated *A. baumannii*. Eales et al. [74] demonstrated that atomic force microscopy (AFM) revealed significant alterations in both the size and surface conformity of *A. baumannii* cells following treatment with peptide concentrations (Bicarinalin and BP100) equal to or surpassing the MBC.

## 3. Materials and Methods

### 3.1. Synthesis, Purification, and Preparation of Peptides

Peptides were prepared by stepwise solid phase synthesis using Fmoc polyamide-active ester chemistry on a Milligen 9050 pepsynthesizer. All Fmoc-amino acids were from Milligen/Bioresearch–Waters (Paris, France). The following compounds were obtained from Milligen/Bioresearch (Paris, France): 4-(Hydroxymethyl) phenoacetic acid-linked polyamide/kieselguhr resin (pepsin kA), Fmoc-aminoacidpentafluorophenyl (Pfp), and 3-hydroxy-2,3-dehydro-4-oxo-benzotriazine (Dhbt) esters. Cleavage of peptidyl-resin and side chain deprotection were carried out using 5 mg of peptidyl-resin in 1 mL of a mixture composed of trifluoroacetic acid, para-cresol, thioanisol, water, and ethyl methyl sulfide (82.5%, 5.5%, and 2.5% (*v*/*v*)) for 2 h at room temperature. After filtering to remove the resin and ether extraction, the crude peptides were purified by a combination of Sephadex gel filtration, ion exchange chromatography, and preparative high performance liquid chromatography (HPLC). The homogeneity of the synthetic peptides was assessed by analytical HPLC, amino acid analysis, solid phase sequence analysis, and mass spectrometry [18]. All peptides were stored frozen as stock solutions at 3.5 mM in double-distilled water at −20 °C.

### 3.2. Calculation of Peptide Physicochemical and Structural Parameters

The properties of our peptides, such as length, net charge (Z) and molecular weight (MW), were calculated using the BACHEM peptide calculator tool. Both hydrophobicity (H) and hydrophobic moment (μH) were calculated using Heliquest software [44]. The total trend of aggregation in aqueous solution was predicted using TANGO software 2.2 [47], while the helicity (α-helix%) of each peptide was calculated using AGADIR software 2s [52]. 

### 3.3. Bacterial Strains and Inoculum Standardization

The bacterial strain used in the present study came from the stock culture of the microbiology laboratory of the Federal University of the Delta of Parnaíba—UFDPar, Parnaíba—PI, Brazil. The strain used in the tests was *A. baumannii* clinical specimen MDR (multidrug resistant), which developed resistance to major antibiotic classes and carbapenem-resistant isolates. Before performing all experiments, the selected strains were cultured in Petri dishes containing Mueller-Hinton agar (Difco™, Piaui, Brazil). Then, under aerobic conditions, they were incubated in a bacteriological oven for 24 h at 35 ± 2 °C. After incubation, the colonies that grew alone were collected with a disposable bacteriological loop and suspended in a sterile saline solution (0.85% NaCl (*w*/*v*), Parnaíba—UFDPar, Piaui, Brazil) in order to reach an absorbance pattern between 0.08 and 0.13 at 625 nm, as tested with a UV-vis spectrophotometer (Shimadzu, Japan), thus corresponding to 0.5 McFarland scale (1–2 × 108 CFU/mL), as recommended by the Clinical Laboratory Standards Institute CLSI [75]. Once standardized, the obtained bacterial suspension was used to prepare the bacterial inoculum used in the execution of MIC determination protocols [75]. 

### 3.4. Antibacterial Experiments

In accordance with the standards recommended by CLSI [75], the antibacterial potential of the peptides was evaluated with the method of determining the MIC, i.e., the broth microdilution method. Using a 96-well microdilution plate (KASVI, Parnaíba, Brazil), the antibacterial effect was analyzed against the bacterial strains. Therefore, 5 μL of each peptide was added to the first line of the microtiter wells containing 195 μL Mueller-Hinton (M-H) broth (Life Technologies, New York, NY, USA), followed by two-fold serial dilutions with final concentrations ranging from 25 µg/mL to 0.19 µg/mL for all peptides. The volume of the bacterial inoculum was equal to 50 µL, which was added to the test wells with the M-H broth at the beginning of the experiment to give a final concentration of 5 × 10^5^ CFU/mL and to reach a final volume of 100 μL in each well after discarding the last additional volume of 100 μL following the serial dilution. MIC was defined as the lowest concentration of an antibacterial agent expressed in μg/mL, which, under strictly controlled in vitro conditions, completely prevents visible bacterial growth after incubation for 24 h at 37 °C in aerobic conditions. The MBC was complementary to the MIC; the MBC demonstrated the lowest concentration of antimicrobial agent that inhibited growth of bacterial colonies on the agar. MBC was verified by seeding 10 μL of the wells that showed results equal to or greater than the MIC onto Mueller-Hinton Agar (MHA)(Life Technologies, New York, USA), with the assistance of a Drigalski spatula. All tests were performed in triplicate. In order to guarantee the quality and safety of the protocols of this study, the manipulation of bacterial strains was performed under aseptic conditions. In addition, all procedures for the execution of the experimental protocols were performed in a class II B2 biological safety cabinet (Buzattos, MG, Parnaíba, Brazil).

### 3.5. Cell Culture

The HEp-2 cell line contained HeLa marker chromosomes and was derived from HeLa contamination. It was obtained from the American Type Culture Collection (ATCC, Manassas, VA, USA). Cells were routinely maintained in a humidified atmosphere of 5% CO_2_ at 37 °C. The Culture Medium Dulbecco’s Modified Eagle Medium (DMEM) (Biofaster, Tunis, Tunisia) was supplemented with 1% L-glutamine, 1% penicillin/streptomycin and 10% (*v*/*v*) heat inactivated fetal bovine serum (FBS)(Biofaster, Tunis, Tunisia).

### 3.6. MTT Assay and Cytotoxicity Analysis

The cytotoxicity test consisted of measuring the viability of cells in culture when they came into contact with the peptides being tested. Cytotoxicity was determined using the 3-(4,5-dimethylthiazol-2-yl)-2,5-diphenyl tetrazolium bromide (MTT) colorimetric assay on cultured cells (HEp-2 lines). The lyophilized peptides were diluted in distilled water to obtain a final concentration of 1 mg/mL. Then, 50% dilutions of the different peptides were then prepared in Eppendorf tubes with the fresh medium DMEM. These concentrations ranged from 100 µg/mL to 1.5 µg/mL. The HEp-2 cells in suspension (MEM medium with 10% FCS) (Biofaster, Tunis, Tunisia) were distributed in 96-well plates at a rate of 100 μL, i.e., containing 10^5^ cells per well, and incubated in an oven at 37 °C under 5% CO_2_ for 24 h. Subsequently, the medium was removed and replaced with 50 μL DMEM medium with 2% FCS. Then, 50 µL of each peptide was added to all wells, and a series of 50% dilutions was carried out from 100 µg/mL to a concentration of 1.5 µg/mL. The test was carried out in triplicate and at three different times. Untreated cells served as a negative control. After 72 h of incubation, the supernatant was collected and the viability of the cells treated with the peptides was determined by the MTT method. Briefly, 50 µL of the MTT solution (5 mg/mL) was added to each well. After 4 h of incubation at 37 °C, the optical density (OD) was measured at 570 nm using an ELISA reader (Multiskan EX, Labsystems, Paris, France), after adding 100 μL of DMSO to dissolve the crystals of formazan. The results were expressed as a percentage of viability relative to the negative control without a peptide, according to the formula: (Viability percentage = (DO_544_ nm peptide/DO_544_ nm control) × 100). The results were expressed as a percentage of viable HEp-2 cells, and the half maximal cytotoxic concentrations to the HEp-2 CC_50_ values were calculated with GraphPad Prism^®^ (version 9.0).

### 3.7. Atomic Force Microscopy (AFM)

With the objective of observing possible morphological alterations caused by the action of an active peptide from the DRS families, bacterial growth control and bacteria treated with K_4_S4 (1-16) were evaluated using Atomic Force Microscopy (AFM). Therefore, the selected bacterial strain, *A. baumannii* MDR, was submitted to this experimental assay. The bacterial inoculum used to obtain the images was 5 × 10^5^ CFU/mL and, for sample preparation, procedures similar to those described by Araújo et al. [73] were used. Briefly, after 24 h of incubation of the MIC determination assay, 20 µL of culture medium from the wells of the treated and untreated (control) groups was deposited on the surface of a glass slide. Then, the samples were submitted to dry in an oven at 37 °C for 10 min. After this time, the samples were carefully washed with distilled water and left to dry under the same conditions described above. After this preparation, AFM images of 6 × 6 µm were obtained using a model TT-AFM microscope (AFM Workshop—Redding, CA, USA) in intermittent contact mode (tapping mode), using TAP300-G10 tips (TED PELLA, INC—Redding, CA, USA) with a resonance frequency of approximately 239 kHz. Multiple areas (n = 10, 0.3 × 0.3 µm) of each sample were examined in order to verify the average roughness of treated and untreated bacteria, using the program Gwyddion 2.60. 

### 3.8. Statistical Analysis

Statistical analyses were performed with the GraphPad Prism 9.0 software (GraphPad Software Inc.) for cell viability assays. The difference between the average roughness (Ra) was statistically analyzed using *t* Test in the GraphPad Prism 8.0 software. Differences were considered as statistically significant at *p* < 0.05.

## 4. Conclusions

In conclusion, the present findings show that our dermaseptines from the family of S4 and B2 have significant and selective antibacterial effects against *Acinetobacter baumannii*. This study also revealed that the cytotoxicity of these modified peptides was concentration dependent. We conclude that the bi-substituted peptide, K_4_K_20_S4, which has the highest CC_50_ (75.71 μg/mL), the highest net positive charge (+6), and the lowest values of MIC and MBC (3.125 μg/mL and 6.25 μg/mL respectively), is the best candidate in terms of antibacterial activity. Collectively, these small molecules may have potential for use as safe antibacterial compounds. Furthermore, the obtained AFM images revealed morphological changes and alterations, as well as increased roughness.

## Figures and Tables

**Table 1:** Structural and physicochemical properties of dermaseptins and their derivatives.

Peptides	Sequence *	Parameters **						
		Length	MW	Net Charge	H	Aggregation	μH	α-Helix%
S4 (Native)	ALWMTLLKKVLKAAAKAALNAVLVGANA	28	2.850	+4	0.544	183.33	0.248	16.55
K4K20S4	ALWKTLLKKVLKAAAKAALKAVLVGANA	28	2.861	+6	0.451	112.02	0.246	11.8
K4S4(1-16)	ALWKTLLKKVLKAAAK	16	1.782	+5	0.426	0	0.526	2.41
B2 (Native)	GLWSKIKEVGKEAAKAAAKAAGKAALGAVSEAV	33	3.181	+3	0.199	9.681	0.204	10.02
K3K4B2	GLKKKIKEVGKEAAKAAAKAAGKAALGAVSEAV	33	3.164	+5	0.072	9.681	0.159	9.85

* The sequences are shown using a one letter code for the amino acids. ** Parameters: MW (kDa); H: Hydrophobicity; Aggregation: total trend of aggregation; μH: Hydrophobic moment; α-Helix%: Helicity.

**Table 2:** Antimicrobial activities and dose-dependent effects of different dermaseptins and their derivatives.

Peptides	CC_50_ Hep-2 Cells (μg/mL)	*A. baumannii* MIC (μg/mL)	*A. baumannii* MBC (μg/mL)
S4	16.51	12.5	25
K_4_S4(1-16)	68.9	6.25	12.5
K_4_K_20_S4	75.71	3.125	6.25
B2	30.4	12.5	25
K_3_K_4_B2	61.25	6.25	12.5
meropenem	ND	32	64

CC_50_: peptide concentration that causes 50% cytotoxicity in HEp-2 cells for dermaseptin S4 and derivatives (μg/mL); ND, not determined; MIC: Minimum Inhibitory Concentration (μg/mL); MBC: Minimal Bactericidal Concentration (μg/mL).

## Data Availability

Materials, data, and associated protocols are available to readers without undue qualifications regarding material transfer agreements. For data retrieval, please contact (email: zairi_amira@yahoo.fr).

## Abbreviations

HAIs	Healthcare-Associated infections
MDR	Multidrug-resistant
MTT	(3-[4,5-dimethylthiazol-2-yl]-2,5 diphenyl tetrazolium bromide)
WHO	World Health Organization
AMPs	Antimicrobial peptides
DRSs	Dermaseptins
AFM	Atomic force microscopy
Pfp	Fmoc-aminoacidpentafluorophenyl
Dhbt	3-hydroxy- 2,3-dehydro-4-oxo-benzotriazine
HPLC	High performance liquid chromatography
MHA	Mueller Hinton Agar
DMEM	Modified Eagle Medium
FBS	Fetal Bovine Serum
Alg NPs	Alginate nanoparticles

## References

[1] Monegro A.F., Muppidi V., Regunath H. (2023). Hospital-Acquired Infections. Treasure Island.

[2] Naveed S., Sana A., Sadia H., Qamar F., Aziz N. (2019). Nosocomial Infection: Causes Treatment and Management. Am. J. Biomed. Sci. Res..

[3] Larypoor M., Frsad S. (2011). Evaluation of nosocomial infections in one of hospitals of Qom, 2008. Iran. J. Med. Microbiol. Persian..

[4] Olise C.C., Simon-Oke I.A. (2018). Fomites: Possible vehicle of nosocomial infections. J. Public Health Catalog..

[5] Houang E.T.S., Sormunen R.T., Lai L., Chan C.Y., Leong A.S.Y. (1998). Effect of desiccation on the ultrastructural appearances of *Acinetobacter baumannii* and *Acinetobacter lwoffii*. J. Clin. Path..

[6] World Health Organization, WHO (2017). Global Antimicrobial Resistance Surveillance System (GLASS), Report: Early Implementation 2016–2017.

[7] Talreja D., Muraleedharan C., Gunathilaka G., Zhang Y., Kaye K.S., Walia S.K., Kumar A. (2014). Virulence properties of multidrug resistant ocular isolates of *Acinetobacter baumannii*. Curr. Eye Res..

[8] Roy S., Chowdhury G., Mukhopadhyay A.K., Dutta S., Basu S. (2022). Convergence of Biofilm Formation and Antibiotic Resistance in *Acinetobacter baumannii* Infection. Front. Med..

[9] Ia K., Diene S.M., Goderdzishvili M., Rolain J.M. (2011). Molecular detection of OXA carbapenemase genes in multidrug-resistant *Acinetobacter baumannii* isolates from Iraq and Georgia. Int. J. Antimicrob. Agents.

[10] Hamidian M., Nigro S.J. (2019). Emergence, molecular mechanisms and global spread of carbapenem-resistant *Acinetobacter baumannii*. Microb. Genom..

[11] Cai Y., Chai D., Wang R., Liang B., Bai N. (2012). Colistin resistance of *Acinetobacter baumannii*: Clinical reports, mechanisms and antimicrobial strategies. J. Antimicrob. Chemoth..

[12] Navon-Venezia S., Leavitt A., Carmeli Y. (2007). High tigecycline resistance in multidrug-resistant *Acinetobacter baumannii*. J. Antimicrob. Chemoth..

[13] Geisinger E., Vargas-Cuebas G., Mortman N.J., Syal S., Dai Y., Wainwright E.L., Lazinski D., Wood S., Zhu Z., Anthony J. (2019). The Landscape of Phenotypic and Transcriptional Responses to Ciprofloxacin in *Acinetobacter baumannii*: Acquired Resistance Alleles Modulate Drug-Induced SOS Response and Prophage Replication. mBio.

[14] Gellings P.S., Wilkins A.A., Morici L.A. (2020). Recent Advances in the Pursuit of an Effective *Acinetobacter baumannii* Vaccine. Pathogens.

[15] Bahar A.A., Ren D. (2013). Antimicrobial peptides. Pharmaceuticals.

[16] Mwangi J., Hao X., Lai R., Zhang Z.Y. (2019). Antimicrobial peptides: New hope in the war against multidrug resistance. Zool. Res..

[17] Brown K.L., Hancock R.E. (2006). Cationic host defense (antimicrobial), peptides. Curr. Opin. Immunol..

[18] Mor A., Nguyen V.H., Delfour A., Migliore-Samour D., Nicolas P. (1991). Isolation, amino acid sequence, and synthesis of dermaseptin, a novel antimicrobial peptide of amphibian skin. Biochemistry.

[19] Amiche M., Ladram A., Nicolas P. (2008). A consistent nomenclature of antimicrobial peptides isolated from frogs of the subfamily Phyllomedusinae. Peptides.

[20] Zairi A., Tangy F., Saadi S., Hani K. (2008). In vitro activity of dermaseptin S4 derivatives against genital infections pathogens. Regul. Toxicol. Pharmacol..

[21] Nicolas P., El Amri C. (2009). The dermaseptin superfamily: A gene-based combinatorial library of antimicrobial peptides. Biochim. Biophys. Acta..

[22] Shai Y. (2002). Mode of action of membrane active antimicrobial peptides. Biopolymers.

[23] Feder R., Dagan A., Mor A. (2000). Structure-activity relationship study of antimicrobial dermaseptin S4 showing the consequences of peptide oligomerization on selective cytotoxicity. J. Biol. Chem..

[24] Amiche M., Ducancel F., Mor A., Boulain J., Menez A., Nicolas P. (1994). Precursors of vertebrate peptide antibiotics dermaseptin b and adenoregulin have extensive sequence identities with precursors of opioid peptides dermorphin, dermenkephalin, and deltorphins. J. Biol. Chem..

[25] Daly J.W., Caceres J., Moni R.W., Gusovsky F., Moos M., Seamon K.B., Milton K., Myers C.W. (1992). Frog secretions and hunting magic in the upper Amazon: Identification of a peptide that interacts with an adenosine receptor. Proc. Natl. Acad. Sci. USA.

[26] Hazime N., Belguesmia Y., Barras A., Amiche M., Boukherroub R., Drider D. (2022). Enhanced Antibacterial Activity of Dermaseptin through Its Immobilization on Alginate Nanoparticles—Effects of Menthol and Lactic Acid on Its Potentialization. Antibiotics.

[27] Galanth C., Abbassi F., Lequin O., Ayala-Sanmartin J., Ladram A., Nicolas P., Amiche M. (2009). Mechanism of antibacterial action of dermaseptin B2: Interplay between helix-hinge-helix structure and membrane curvature strain. Biochemistry.

[28] Gautier R., Douguet D., Antonny B., Drin G. (2008). HELIQUEST: A web server to screen sequences with specific α-helical properties. Bioinformatics.

[29] Fernández-Escamilla A.M., Rousseau F., Schymkowitz J., Serrano L. (2004). Prediction of sequence-dependent and mutational effects on the aggregation of peptides and proteins. Nat. Biotec..

[30] Muñoz V., Serrano L. (1994). Elucidating the folding problem of helical peptides using empirical parameters. Nat. Struc. Mol. Bio..

[31] Clinical and Laboratory Standards Institute (2018). CLSI Performance Standards for Antimicrobial Susceptibility Testing; Approved Standard—28th ed M100.

[32] de Araujo A.R., Quelemes P.V., Perfeito M.L., de Lima L.I., Sá M.C., Nunes P.H., Joanitti G.A., Eaton P., Soares M.J., de Souza de Almeida Leite J.R. (2015). Antibacterial, antibiofilm and cytotoxic activities of *Terminalia fagifolia* Mart. extract and fractions. Ann. Clin. Microbiol. Antimicrob..

[33] Efron L., Dagan A., Gaidukov L., Ginsburg H., Mor A. (2002). Direct interaction of dermaseptin S4 aminoheptanoyl derivate with intraerythrocytic malaria parasite leading to increased specific antiparasitic activity in culture. J. Biol. Chem..

[34] Kustanovich I., Shalev D.E., Mikhlin M., Gaidukov L., Mor A. (2002). Structural requirements for potent versus selective cytotoxicity for antimicrobial dermaseptin S4 derivatives. J. Biol. Chem..

[35] Ong Z.Y., Wiradharma N., Yang Y.Y. (2014). Strategies employed in the design and optimization of synthetic antimicrobial peptide amphiphiles with enhanced therapeutic potentials. Adv. Drug. Deliv..

[36] Ma Z., Wei D., Yan P., Zhu X., Shan A., Bi Z. (2015). Characterization of cell selectivity, physiological stability and endotoxin neutralization capabilities of alpha-helix-based peptide amphiphiles. Biomaterials.

[37] Lyu Y., Yang Y., Lyu X., Na D., Shan A. (2016). Antimicrobial activity, improved cell selectivity and mode of action of short PMAP-36-derived peptides against bacteria and Candida. Sci. Rep..

[38] Dong N., Zhu X., Chou S., Shan A., Li W., Jiang J. (2014). Antimicrobial potency and selectivity of simplified symmetric-end peptides. Biomaterials.

[39] Van Zoggel H., Carpentier G., Dos Santos C., Hamma-Kourbali Y., Courty J., Amiche M., Delbé J. (2012). Antitumor and angiostatic activities of the antimicrobial peptide dermaseptin B2. PLoS ONE.

[40] Irazazabal L.N., Porto W.F., Ribeiro S.M., Casale S., Humblot V., Ladram A., Franco O.L. (2016). Selective amino acid substitution reduces cytotoxicity of the antimicrobial peptide mastoparan. Biochim. Biophys. Acta.

[41] Navon-Venezia S., Feder R., Gaidukov L., Carmeli Y., Mor A. (2002). Antibacterial properties of dermaseptin S4 derivatives with in vivo activity. Antimicrob. Agents Chemother..

[42] Krugliak M., Feder R., Zolotarev V.Y., Gaidukov L., Dagan A., Ginsburg H., Mor A. (2000). Antimalarial activities of dermaseptin S4 derivatives. Antimicrob. Agents Chemother..

[43] Walter R., Neidle A., Marks N. (1975). Significant differences in the degradation of pro-leu-gly-nH2 by human serum and that of other species (38484). Proc. Soc. Exp. Biol. Med..

[44] Hong S.Y., Oh J.E., Lee K.H. (1999). Effect of D-amino acid substitution on the stability, the secondary structure, and the activity of membrane-active peptide. Biochem. Pharmacol..

[45] Braunstein A., Papo N., Shai Y. (2004). In vitro activity and potency of an intravenously injected antimicrobial peptide and its DL amino acid analog in mice infected with bacteria. Antimicrob. Agents Chemother..

[46] Zhao Y., Zhang M., Qiu S., Wang J., Peng J., Zhao P., Zhu R., Wang H., Li Y., Wang K. (2016). Antimicrobial activity and stability of the D-amino acid substituted derivatives of antimicrobial peptide polybia-MPI. AMB. Express.

[47] Vaezi Z., Bortolotti A., Luca V., Perilli G., Mangoni M.L., Khosravi-Far R., Bobone S., Stella L. (2020). Aggregation determines the selectivity of membrane-active anticancer and antimicrobial peptides: The case of killer FLIP. Biochim. Biophys. Acta Biomembr..

[48] Al Musaimi O., Valenzo O.M.M., Williams D.R. (2023). Prediction of peptides retention behavior in reversed-phase liquid chromatography based on their hydrophobicity. J. Sep. Sci..

[49] Eisenberg D., Weiss R.M., Terwilliger T.C. (1984). The hydrophobic moment detects periodicity in protein hydrophobicity. Proc. Nat. Acad. Sci. USA.

[50] Eisenberg D., Weiss R.M., Terwilliger T.C. (1982). The helical hydrophobic moment: A measure of the amphiphilicity of a helix. Nature.

[51] Dennison S.R., Phoenix D.A. (2011). Influence of C-terminal amidation on the efficacy of modelin-5. Biochemistry.

[52] Bartels E.J.H., Dekker D., Amiche M. (2019). Dermaseptins, Multifunctional Antimicrobial Peptides: A Review of Their Pharmacology, Effectivity, Mechanism of Action, and Possible Future Directions. Front. Pharmacol..

[53] Zou R., Zhu X., Tu Y., Wu J., Landry M.P. (2018). Activity of Antimicrobial Peptide Aggregates Decreases with Increased Cell Membrane Embedding Free Energy Cost. Biochemistry.

[54] Torres M., Sothiselvam S., Lu T.K., de la Fuente-Nunez C. (2019). Peptide Design Principles for Antimicrobial Applications. J. Mol. Biol..

[55] Huang Y., He L., Li G., Zhai N., Jiang H., Chen Y. (2014). Role of helicity of α-helical antimicrobial peptides to improve specificity. Prot. Cell.

[56] Zelezetsky I., Tossi A. (2006). Alpha-helical antimicrobial peptides—Using a sequence template to guide structure–activity relationship studies. Bioch. Biophy. Acta.

[57] Belaid A., Aouni M., Khelifa R., Trabelsi A., Jemmali M., Hani K. (2002). In vitro antiviral activity of dermaseptins against herpes simplex virus type 1. J. Med. Virol..

[58] Gourkhede D.P., Bhoomika S., Pathak R., Yadav J.P., Nishanth D., Vergis J., Malik S.V.S., Barbuddhe S.B., Rawool D.B. (2020). Antimicrobial efficacy of Cecropin A (1–7)-Melittin and Lactoferricin (17–30) against multi-drug resistant *Salmonella Enteritidis*. Microb. Pathog..

[59] Sruthy K.S., Nair A., Antony S.P., Puthumana J., Singh I.S.B., Philip R. (2019). A histone H2A derived antimicrobial peptide, Fi-Histin from the Indian White shrimp, Fenneropenaeus indicus: Molecular and functional characterization. Fish Shellfish Immunol..

[60] Zairi A., Serres C., Tangy F., Jouannet P., Hani K. (2008). In vitro spermicidal activity of peptides from amphibian skin: Dermaseptin S4 and derivatives. Bioorg. Med. Chem..

[61] Belaid A., Braiek A., Alibi S., Hassen W., Beltifa A., Nefzi A., Mansour H.B. (2021). Evaluating the effect of dermaseptin S4 and its derivatives on multidrug-resistant bacterial strains and on the colon cancer cell line SW620. Environ. Sci. Pollut. Res. Int..

[62] Lorin C., Saidi H., Belaid A., Zairi A., Baleux F., Hocini H., Bélec L., Hani K., Tangy F. (2005). The antimicrobial peptide dermaseptin S4 inhibits HIV-1 infectivity in vitro. Virology.

[63] Streit F., Perl T., Schulze M.H., Binder L. (2016). Personalised beta-lactam therapy: Basic principles and practical approach. Lab. Med..

[64] (2007). MerremRM, IV (Meropenem for Injection): US Prescribing Information.

[65] Steffens N.A., Zimmermann E.S., Nichelle S.M., Brucker N. (2021). Meropenem use and therapeutic drug monitoring in clinical practice: A literature review. J. Clin. Pharm. Ther..

[66] Jiang Z., Vasil A.I., Vasil M.L., Hodges R.S. (2014). “Specificity Determinants” Improve Therapeutic Indices of Two Antimicrobial Peptides Piscidin 1 and Dermaseptin S4 Against the Gram-negative Pathogens *Acinetobacter baumannii* and *Pseudomonas aeruginosa*. Pharmaceuticals.

[67] Strahilevitz J., Mor A., Nicolas P., Shai Y. (1994). Spectrum of antimicrobial activity and assembly of dermaseptin-b and its precursor form in phospholipid membranes. Biochemistry.

[68] Zairi A., Ferrieres L., Latour-Lambert P., Beloin C., Tangy F., Ghigo J.M., Hani K. (2014). In vitro activities of dermaseptins K4S4 and K4K20S4 against *Escherichia coli*, *Staphylococcus aureus*, and *Pseudomonas aeruginosa* planktonic growth and biofilm formation. Antimicrob. Agents Chemother..

[69] Liu J., Wu Q., Li L., Xi X., Wu D., Zhou M., Chen T., Shaw C., Wang L. (2017). Discovery of phylloseptins that defense against gram-positive bacteria and inhibit the proliferation of the non-small cell lung cancer cell line, from the skin secretions of Phyllomedusa frogs. Molecules.

[70] Zhu H., Ding X., Li W., Lu T., Ma C., Xi X., Wang L., Zhou M., Burden R., Chen T. (2018). Discovery of two skin derived dermaseptins and design of a TAT-fusion analogue with broad-spectrum antimicrobial activity and low cytotoxicity on healthy cells. PeerJ.

[71] Santajit S., Indrawattana N. (2016). Mechanisms of Antimicrobial Resistance in ESKAPE Pathogens. Biomed. Res. Int..

[72] O’Shea P. (2003). Intermolecular interactions with/within cell membranes and the trinity of membrane potentials: Kinetics and imaging. Biochem. Soc. Trans..

[73] Lin B., Hung A., Li R., Barlow A., Singleton W., Matthyssen T., Sani M.A., Hossain M.A., Wade J.D., O’Brien-Simpson N.M. (2022). Systematic comparison of activity and mechanism of antimicrobial peptides against nosocomial pathogens. Eur. J. Med. Chem..

[74] Pouny Y., Rapaport D., Mor A., Nicolas P., Shai Y. (1992). Interaction of antimicrobial dermaseptin and its fluorescently labeled analogues with phospholipid membranes. Biochemistry.

[75] Eales M.G., Ferrari E., Goddard A.D., Lancaster L., Sanderson P., Miller C. (2018). Mechanistic and phenotypic studies of bicarinalin, BP100 and colistin action on *Acinetobacter baumannii*. Res. Microbiol..

