# Correction: Haddad et al. Evaluation of the Antibacterial Activity of New Dermaseptin Derivatives against *Acinetobacter baumannii*. *Pharmaceuticals* 2024, *17*, 171

**DOI:** 10.3390/ph17091146

**Published:** 2024-08-30

**Authors:** Houda Haddad, Radhia Mejri, Amira Zaïri

**Affiliations:** 1 BIOLIVAL Laboratory, LR14ES06, The Higher Institute of Biotechnology of Monastir ISBM, University of Monastir, Monastir 5000, Tunisia; haddadhouda1993@gmail.com; 2 Biochemistry Department, LR18ES47, Faculty of Medicine, University of Sousse, Sousse 4002, Tunisia; mejriradhia116@gmail.com

**Removal of an Author**

**Alyne Rodrigues de Araujo** requested to remove his name from the original publication [1] because he did not participate in the analysis of the original data presented in this paper. The corrected Author Contributions statement appears here. The authors state that the scientific conclusions are unaffected. This correction was approved by the Academic Editor. The original publication has also been updated.

**Author Contributions:** This manuscript is the overall collaborative work of the participants. Writing, Methodology, Conceptualization, H.H.; Review-Validation, R.M.; Supervision, Review & Editing, A.Z. All authors have read and agreed to the published version of the manuscript.
